# Test–retest reliability of splenic volume assessment by ultrasonography

**DOI:** 10.1038/s41598-022-23384-6

**Published:** 2022-11-08

**Authors:** Pontus Holmström, Frank Pernett, Erika Schagatay

**Affiliations:** 1 Environmental Physiology Group, Department of Health Sciences, Mid Sweden University, Kunskapsgatan 4, 83140 Östersund, Sweden; 2 Swedish Winter Sports Research Centre, Department of Health Sciences, Mid Sweden University, Östersund, Sweden

**Keywords:** Physiology, Gastrointestinal system

## Abstract

While MRI and CT are the gold standards for assessments of splenic size in clinical settings, ultrasonography is particularly suited due to its portability, cost efficiency and easy utilization. However, ultrasonography is associated with subjective assessment, potentially resulting in increased variation. We used a test–retest design aiming to determine the reliability of splenic measurements assessed by ultrasonography during apnea. In addition, we compared reliability between different equations for volume calculations: Koga, Prolate ellipsoid and Pilström. Twelve healthy participants (6 women) performed two tests separated by 15 min, comprising a maximal voluntary apnea in a seated position. Splenic dimensions were measured via ultrasonography for 5 min before and immediately following apnea. Resting splenic volume displayed high test–retest reliability between tests (Pilström: 157 ± 39 mL vs 156 ± 34 mL, *p* = .651, ICC = .970, *p* < .001, CV = 2.98 ± 0.1%; Prolate ellipsoid: 154 ± 37 mL vs 144 ± 43 mL, *p* = .122, ICC = .942, *p* < .001, CV = 5.47 ± 0.3%; Koga: 142 ± 37 mL vs 140 ± 59 mL, *p* = .845, ICC = .859, *p* < .001, CV = 9.72 ± 1.4%). Apnea-induced volumes displayed similar reliability (127 ± 29 mL vs 129 ± 28 mL, *p* = .359, ICC = .967, *p* < .001, CV = 3.14 ± 3.1%). Reliability was also high between equations (Pilström vs Prolate ellipsoid: ICC = .818, *p* < .001, CV = 7.33 ± 0.3%, bias =  − 3.1 mL, LoA =  − 46.9 to 40.7 mL; Pilström vs Koga: ICC = .618, *p* < .01, CV = 11.83 ± 1.1%, bias =  − 14.8 mL, LoA =  − 76.9 to 47.3 mL). We conclude that splenic ultrasonographic measurements have practical applications during laboratory and field-based research as a reliable method detecting splenic volume change consistently between repeated tests. The Pilström equation displayed similar reliability compared to the prolate ellipsoid formula and slightly higher compared to the Koga formula and may be particularly useful to account for individual differences in splenic dimensions.

## Introduction

The spleen is a highly vascularized organ with a continuous blood flow pulsating through it^1^, which is important to initiate immune responses^2^. In addition, it stores ~ 10% of the body’s total red blood cells (RBC)^3^, which through contraction of smooth muscles^4^, can be expelled into the systemic circulation^5–9^, thereby enhancing oxygen carrying-capacity. There is great individual variation associated with splenic size, both in measures of specific diameters (maximal length, width and thickness)^10^ and in resting volume, ranging from 58 to 375 mL in healthy individuals^11–13^.

Determination of normative splenic volumes is particularly important in medical work, to accurately diagnose, for example, splenomegaly^13^ and various hematological diseases. In such clinical settings, helical computed tomography (CT) and magnetic resonance (MR) imaging are the gold standard methods^14,15^. However, these methods are also stationary and expensive. On the other hand, ultrasonography is a useful non-invasive, portable method, which is applicable in both laboratory settings and during fieldwork contexts. Ultrasonic imaging is particularly suited to experiments involving various stressors, such as maximal exercise^3,16^, voluntary apnea^8,17^, normobaric hypoxia^18^ or hypobaric hypoxia^19^, which often includes a time-constrained protocol where windows for collection of splenic measurements are short. The length of the spleen display a strong correlation with splenic volumes from measures of cadavers^20^ and with CT scan measurements^14,21^.

The first model to compute splenic volume measures based on ultrasonpgraphy was designed by Koga^22,23^, who formulated an equation based on measures of maximal splenic length and thinckness yielding a measure of sectional area. More recent research, however, uses computation of maximal splenic length, width and thickness, to estimate the volume, whereby a prolate ellipsoid formula is considered the general convention^11,13,14,24^. Nonetheless, other formulas exist, one, in particular, was designed to account for individual differences in splenic diameters, which could plausibly result in varying splenic volumes. This particular formula has been broadly used in physiological laboratory research, involving apneic diving^8,25,26^, normobaric hypoxia exposure^18,27–29^ and during maximal exercise^16^. Additionally, it has been used during field-based contexts involving hypobaric hypoxia and exercise^17,19,30,31^. However, the reliability of ultrasonography-derived splenic volumes between repeated measures using this particular formula is to date unknown.

Determination of reliability of splenic volume assessed by ultrasound is important in physiological research, wherein the focus is towards assessment of the magnitude of a response induced by a specific stressor, e.g. hypoxia, apnea or maximal exercise. Therefore, the ability to replicate measurements consistently over time on repeated measurements, with the highest possible accuracy is important to be able to draw valid conclusions. To the best of our knowledge, research assessing the reliability of splenic volumes by ultrasonography is limited. Hosey et al.^10^ reported moderate to good intra-rater reliability and good inter-rater reliability between repeated tests, however not on actual splenic volume measures or in response to apnea. Accordingly, by adopting a test–retest design, we firstly aimed to determine the absolute and relative reliability of splenic volume, assessed by ultrasonography at baseline and immediately after maximal voluntary apnea. Secondly, we aimed to determine the reliability of different calculation methods that are used to establish a baseline splenic volume, and thirdly, it was an aim to compare reliability measures of the Pilström formula with two other widely accepted formulas for splenic volume calculations.

## Methods

###  Participants recruitment

12 healthy participants, inexperienced in apneic diving, volunteered for the investigation (six females and six males; age: 30 ± 6 yrs; height: 170 ± 6 cm; body mass: 70 ± 11 kg: body mass index: 24.4 ± 3 kg/m^2^). The participants were recruited via convenience sample from the department of health science at Mid Sweden University, either by answering official adverts at the department or by word a mouth. Sample size conciderations were based on a required power of 0.8 and an alpha level fixed at 0.05 yeilding a required sample size of mininimum10 participants in order to detect an intraclass correlation coefficient (ICC) of 0.8 and higher, given two observations per participant^32^. These conciderations were made on the grounds that we expected splenic volume to exhibit low variations between tests, which we have found in previous pilot attempts in our laboratory, as such with lower variation a smaller sample would typically be requited to detect higher ICC. Only one rater collected the measurments.

### Study overview

**Figure 1:**
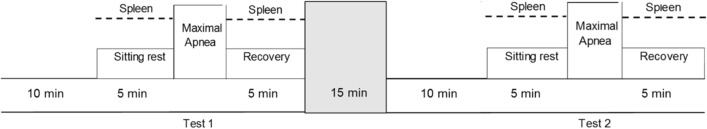
Experimental protocol for test 1 and test 2. Both tests were identical and started with 10 min of lying (supine) rest, after which participants were moved to a sitting 5-min rest, at which splenic measurements were collected each minute for baseline (resting) splenic volume assessment. Thereafter, the maximal voluntary apnea was performed and immediately on termination, a 5-min resting recovery period followed, wherein splenic measurements were collected for apnea-induced volume assessments. Test 1 and test 2 were separated by 15 min, during which the participants were cleared of all testing equipment and asked to leave the laboratory, to return when test 2 should start.

The experiment comprised a test–retest design, consisting of two apnea tests (test 1 and test 2, Fig. 1), which were separated by 15 min. On termination of test 1, all equipment was detached and the ultrasound gel cleaned off, after which the participants were asked to stand and leave the laboratory while walking around. On return to the laboratory, test 2 commenced in the same manner as test 1. This design was chosen, aiming to exclude any factors that may result in temporary splenic changes (i.e. food intake, exercise, fasting, low O_2_ saturation or infections), and possibly reduce unwanted variation. Participants were instructed to abstain from alcohol for at least 24 h before testing and from caffeine or tobacco on the day of the trial before testing. After height (cm) and body mass (kg) were measured (Seca 764, Hamburg, Germany), participants rested in a supine position for 10 min.

### Apnea tests

The apnea test consisted of one static apnea of maximal voluntary duration in air, which was performed in a sitting position and preceded by spontaneous breathing that started after a coached inspiration on the request to take a deep, but not maximal breath. This technique generally results in inhalation of approximately 85% vital capacity^33^. Participants were notified when two and one minutes remained to initiate the breath-hold, when 30 s remained they were handed a nose clip and when 10 s remained a second-by-second countdown started. Participants were asked to refrain from hyperventilation before the apnea. If SpO_2_ (peripheral oxygen saturation) fell below 65%, the participants were told to terminate the apnea and resume breathing, to avoid the risk of hypoxic syncope. Upon termination of apnea, the nose clip was removed, and the participants were instructed to resume normal breathing.

### Splenic ultrasound measurements

**Figure 2:**
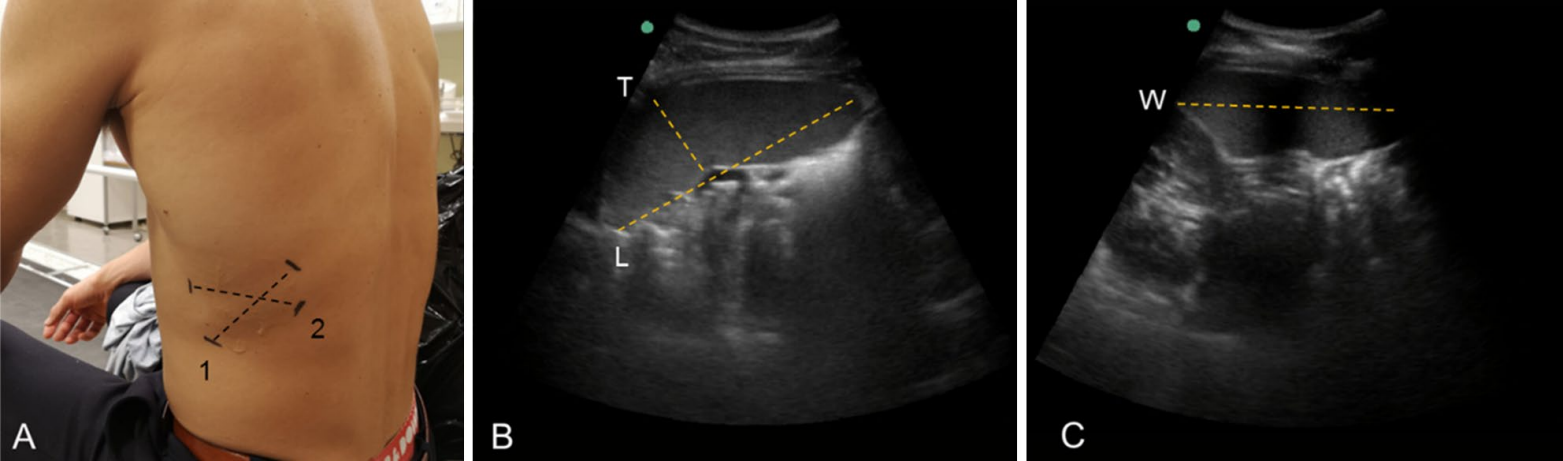
Determination of splenic size in resting state with ultrasonography, which was collected on the dorsal side of the body (**A**). Representative ultrasound images are shown to illustrate the measurement of maximal splenic length (L) and thickness (T) in the longitudinal plane (**B**) and line 1 in (**A**) and width (W) in the transverse plane (**C**) and line 2 in (**A**).

Participants were seated on a chair while the spleen site was identified on the dorsal side of the body (Fig. 2). The splenic size was measured via ultrasonic imaging (M-Turbo Ultrasound system, FUJIFILM Sonosite Inc, Bothell, WA USA) with the probe: C60x/5–2 MHz (SonoSite Inc, Bothell, WA, USA), by an experienced sonographer (PH) and measurements were collected each minute for five minutes during the rest period before the apnea, and immediately after termination of the apnea, during which measurements were collected each minute until five minutes after the apnea (Fig. 1). Two pictures were recorded each minute for determination of the three-axial maximal diameters of the spleen: length, thickness and width, and analyzed in real-time from still images (Fig. 2).

### Analysis

The three-axial maximal diameters of splenic length (L), thickness (T) and width (W) were then used to calculate splenic volume according to the following equations:

$$Pilstr\"o m\;formula\;\left( 1 \right):\;V_{spleen} = \left( {{\text{L}}\uppi \left( {{\text{WT}} - {\text{T}}^{{2}} } \right)/{3}} \right).$$

The equation is based on the observed average shape of the spleen detected on the ultrasonic images^8^, and have been used elsewhere in similar experiments involving splenic volume assessments^17–19,25,30^.

$$Prolate\;ellipsoid\;formula\left ( 2 \right):\;V_{spleen} = \left( {{\text{L}} \times {\text{T}} \times {\text{W}} \times 0.{524}} \right)$$

The equation describes rotation about the ellipses major axis, resulting in a prolate, which has been broadly used in physiological and clinical research^11,13,14,24^.

$$Koga\;formula\;\left( 3 \right):\;V_{spleen} = \left( {{7}.{\text{53S}}{-}{77}.{56}} \right)$$

This equation uses the sectional area (S), to estimate a representative volume measure^23^, which includes measurements of maximal splenic length and thickness^22^.

The standard approach for establishing a baseline splenic volume in this study uses the individual measurements obtained from the five minutes before the apnea, by averaging the ‘two consecutive maximal measurement’ values. This particular method was implemented to limit any influence of variability that may occur due to pulsatile changes in splenic volume and minor changes in probe placement^17,25^. In addition, to compare reliability measures of different approaches of establishing baseline volume, a ‘mean of all measures’ were also computed, simply as an average of all splenic measures obtained from the five minutes before the apnea and ‘one single measure’. The single measure was taken as the value one minute before the apnea, to account for possible anticipatory-induced splenic contractions.

### Statistical analysis

Data are presented as mean ± standard deviation (SD) and were analyzed using IBM SPSS 24.0 for Windows (SPSS Inc., Chicago, IL). Shapiro–Wilks and Kolmogorov–Smirnov tests (*p* > .05) were used to assess if study variables were normally distributed. Paired samples t-tests were used to assess the difference in splenic volume between test 1 and test 2, as well as between formulas. To assess the relative reliability of splenic volumes between test 1 and test 2, and between formulas, ICCs were used, designed as a two-way mixed effect model with absolute agreement, which was considered appropriate to assess reliability with repeated measures including one rater^34^. The level of reliability was determined based on ICC: ICC less than 0.5 was considered poor reliability, ICC between 0.5 and 0.75 indicated moderate reliability, ICC values between 0.75 and 0.9 indicated good reliability and ICCs greater than 0.9 indicated excellent reliability^34^. ICCs were presented with 95% confidence intervals (95% CI). The coefficient of variation (CV) was calculated as the SD of the individual mean value of test 1 and test 2, after which the square root of the mean CV squared was calculated. Typical error (TE) was calculated as the SD of the difference between test 1 and test 2 divided by the square root. To further assess the agreement of splenic volumes, calculated by three different formula, Bland and Altman plots with associated limits of agreement (95% CI from − 1.96 SD to + 1.96 SD) was used as described by Giavarina^35^.

### Ethics approval

The study abided by the regional ethical review board in Umeå, Sweden (reference: 2018-46-31M) and the Declaration of Helsinki. In addition, all measurments were non-invasive, thereby the experiment design did not expose the participants to any increased risks.

## Results

**Figure 3:**
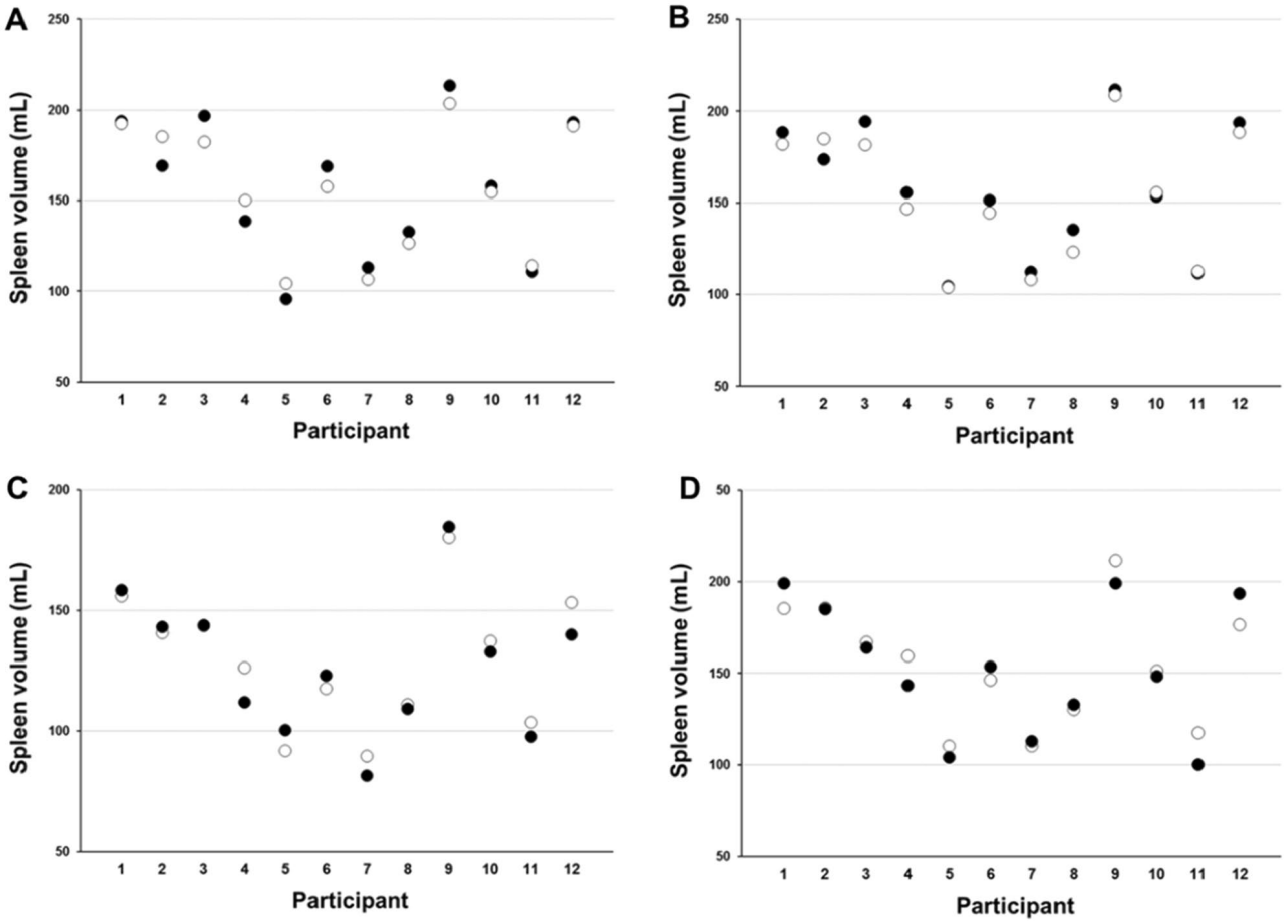
Individual splenic volumes for test 1 (black dots) and test 2 (white dots) for baseline (resting) splenic volume as calculated by ‘two consecutive maximal measures’ (mL; **A**), baseline splenic volume as calculated by ‘mean of all measures’ (mL; **B**), apnea-induced splenic volume and (mL; **C**) after five minutes of recovery (mL; **D**) for each participant using formula (1) (n = 12).

Baseline splenic volume displayed high reliability between tests for ‘two consecutive maximal measures’ (ICC = .970, *p* < .001, Table 1 and Fig. 3A) and ‘mean of all measures’ (ICC = .979, *p* < .001, Table 1 and Fig. 3B), and reliability was reduced for ‘one single measure’ (ICC = .850, *p* < .001, Table 1). Apnea induced-splenic volume also displayed high reliability for ‘two consecutive maximal measures’ (ICC = .967, 95% CI [.894 to .990], *p* < .001, Fig. 3C), with corresponding CV of 3.14 ± 3.1% and TE of 4.9 mL. Splenic volume was significantly reduced immediately after apnea in both tests (*p* < .001), to 127 ± 29 mL (− 18.0%) in test 1 and 129 ± 28 mL (− 16.5%) in test 2 (mean difference of 2.0 mL, 95% CI [− 6.6 to 2.6], *p* = .359). After five minutes of recovery, splenic volume had reached baseline values in both tests (*p* > .05), to 153 ± 36 mL in test 1 and 154 ± 33 mL in test 2 (mean difference of 1.2 mL, 95% CI [− 8.1 to 5.6], *p* = .706; ICC = .954, 95% CI [.849 to .986], *p* < .001, Fig. 3D) and a corresponding CV of 3.57 ± 0.2% and a TE of 7.3 mL.

**Table 1:** Comparisons of three different methods of establishing baseline splenic volume (Mean ± SD) between repeated tests using different formulas with corresponding reliability measures.

	Test 1	Test 2	*p* value	CV (%)	TE (mL)	ICC	95% CI
**Pilström formula (1)**							
Two consecutive maximal measures (mL)	157 ± 39	156 ± 34	.651	2.98 ± 0.1	6.4	.970***	.900 to .991
Mean of all measures (mL)	157 ± 35	153 ± 34	.068	2.37 ± 0.1	4.5	.979***	.915 to .994
One single measure (mL)	161 ± 38	155 ± 37	.308	6.82 ± 0.6	14.3	.850***	.577 to .954
**Prolate ellipsoid formula (2)**							
Two consecutive maximal measures (mL)	154 ± 37	144 ± 43	.122	5.47 ± 0.3	8.6	.942***	.808 to .983
Mean of all measures (mL)	149 ± 36	148 ± 43	.722	4.27 ± 0.2	8.4	.954***	.850 to .987
One single measure (mL)	158 ± 43	149 ± 42	.133	7.52 ± 0.5	13.8	.879***	.619 to .962
**Koga formula (3)**							
Two consecutive maximal measures (mL)	142 ± 38	140 ± 59	.845	9.72 ± 1.4	17.6	.859***	.580 to .958
Mean of all measures (mL)	142 ± 39	142 ± 49	.997	7.07 ± 0.5	13.8	.911***	.718 to .973
One single measure (mL)	152 ± 49	143 ± 48	.241	9.25 ± 1.0	15.6	.892***	.681 to .967

***p < .001.

**Figure 4:**
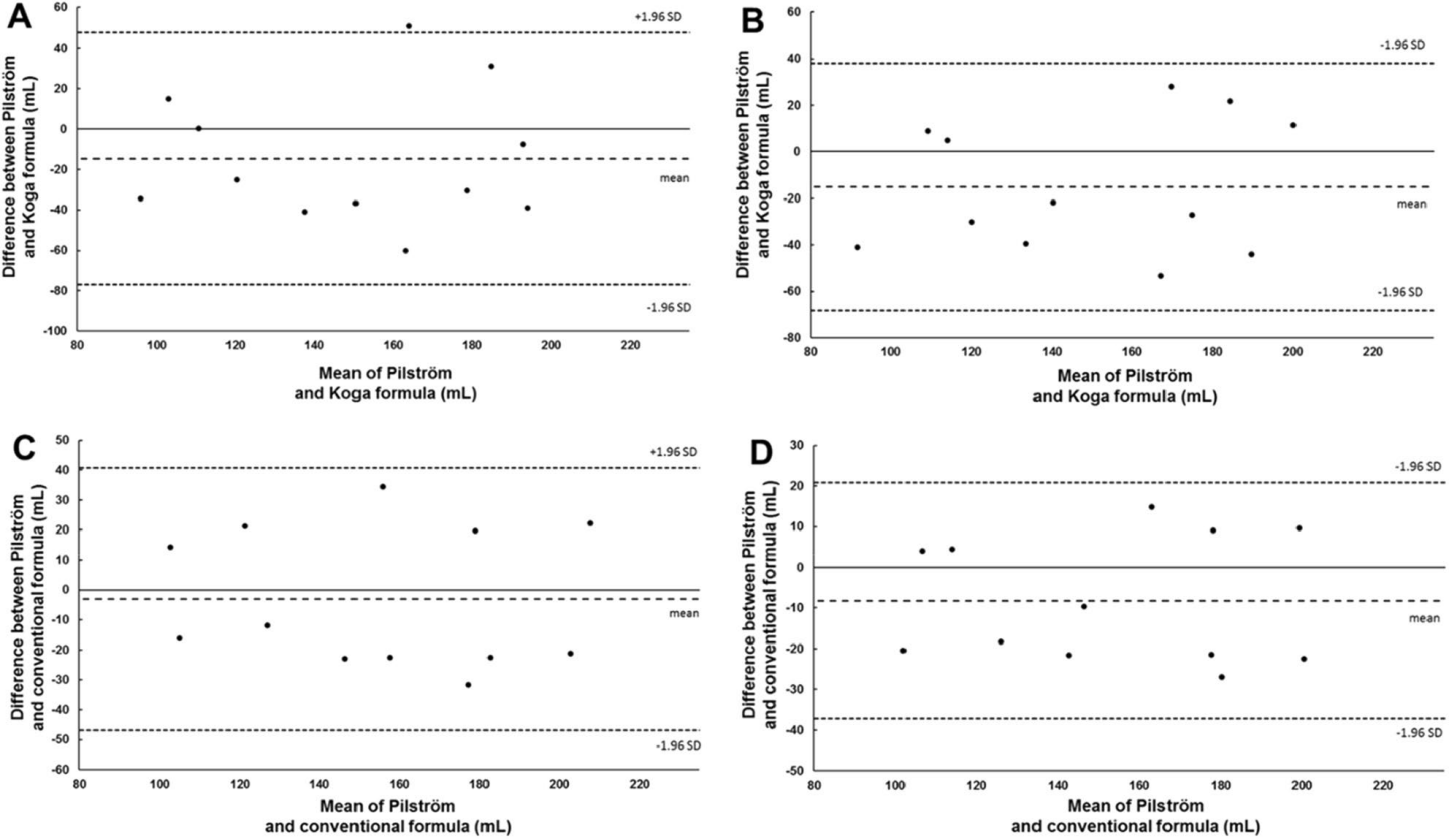
Bland and Altman plot for baseline splenic volume measures. The upper panel displays splenic volume difference (mL; Y-axis) between, and mean splenic volume (mL; X-axis) of formula (1) and formula (3) using ‘two consecutive maximal measures’ (**A**) and ‘mean of all measures’ (**B**). The lower panel displays splenic volume difference (mL; Y-axis) between, and mean splenic volume (mL; X-axis) of formula (1) and the formula (2) using ‘two consecutive maximal measures’ (**C**) and ‘mean of all measures’ (**D**). The bias is represented (middle dotted line) along with zero difference (solid line) with the representation of the upper and lower limits of agreements (small dotted lines), from − 1.96 SD to + 1.96 SD (n = 12).

Formula (1) displayed similar levels of reliability compared to formula (2) for all volume calculation methods, whereas formula (3) displayed lower reliability for each calculation method (Table 1). The reliability between each formula is reported in Tables 2 and 3. The agreement between formula (1) and formula (3) as assessed by Bland and Altman plots, displayed a bias (systemtic measurement error) of − 14.8 mL (95% CI [5.4 to − 34.9]), with the upper 95% limits of agreement of 47.3 mL and the lower 95% limits of agreements of − 76.9 mL (Fig. 4A) for ‘two consecutive maximal measures’ and a bias of − 15.2 mL (95% CI [2.1 to − 32.4]), with the upper 95% limits of agreement of 38.1 mL and the lower 95% limits of agreements of − 68.4 mL (Fig. 4B) for ‘mean of all measures’. The agreement between formula (1) and formula (2) displayed a bias of − 3.1 mL (95% CI [11.1 to − 17.3]), with the upper 95% limits of agreement of 40.7 mL and the lower 95% limits of agreements of − 46.9 mL (Fig. 4C) for ‘two consecutive maximal measures’ and a bias of − 8.2 mL (95% CI [1.1 to − 17.6]), with the upper 95% limits of agreement of 20.7 mL and the lower 95% limits of agreements of − 37.2 mL (Fig. 4D) for ‘mean of all measures’.

**Table 2:** Comparisons of different methods of establishing baseline splenic volume (Mean ± SD) between Pilström and Koga formulas with corresponding reliability measures.

	Pilström formula (1)	Koga formula (3)	*p* value	CV (%)	TE (mL)	ICC	95% CI
Two consecutive maximal measures (mL)	157 ± 39	142 ± 38	.150	11.83 ± 1.1	22.4	.618**	.138 to .870
Mean of all measures (mL)	157 ± 36	142 ± 39	.091	11.24 ± 1.4	19.9	.693**	.242 to .900
One single measure (mL)	161 ± 38	152 ± 49	.377	12.59 ± 1.3	15.2	.671**	.205 to .892

**p < .01. Values refer to comparisons of test 1.

**Table 3:** Comparisons of different methods of establishing baseline splenic volume (Mean ± SD) between Pilström and Conventional formulas with corresponding reliability measures.

	Pilström formula (1)	Prolate ellipsoid formula (2)	*p* value	CV (%)	TE (mL)	ICC	95% CI
Two consecutive maximal measures (mL)	157 ± 39	154 ± 37	.654	7.33 ± 0.3	15.8	.818***	.485 to .944
Mean of all measures (mL)	157 ± 36	149 ± 36	.092	5.67 ± 0.3	10.4	.892***	.652 to .968
One single measure (mL)	161 ± 38	158 ± 43	.586	6.96 ± 0.3	15.2	.857***	.583 to .957

***p < .001. Values refer to comparisons of test 1.

**Figure 5:**
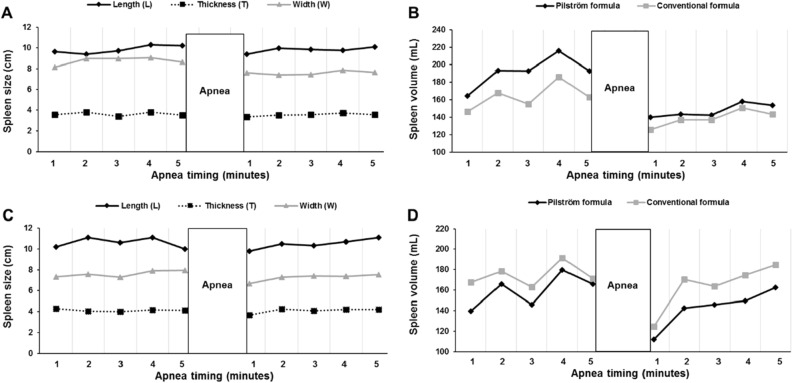
Individual variation in splenic volume as a function of variations in splenic diameters. Panel (**A**) and (**C**) display the size of each splenic diameter (length, thickness and width; cm) and panels (**B**) and (**D**) display splenic volume (mL), calculated by formula (1) (black line) and formula (2) (grey line) from the measures in panel (**A**) and (**C**), for two participants [nr 1 (**A, B**) and nr 2 (**C**, **D**)]. The apnea test started with five minutes of sitting rest, during which splenic measurements (**A**, **C**) were collected each minute for determination of baseline volume (**B**, **D**), following one maximal voluntary apnea. Thereafter, an additional five minutes of rest followed, during which splenic measures (**A**, **C**) were collected each minute, for apnea-induced contraction and recovery volume (**B**, **D**).

The two-volume measures which were used to determine baseline splenic volume through the ‘two consecutive maximal measures’ method displayed CV of 2.23 ± 0.04% (TE = 4.8 mL) and 1.14 ± 0.01% (TE = 2.0 mL) for test 1 and test 2 respectively. Figure 5 illustrates the individual variation in splenic volume values in response to apnea between formula (1) and formula (2), as a function of varying splenic diameters.

## Discussion

This study aimed to determine the test–retest reliability of splenic volume assessed by ultrasonography at baseline and in response to maximal voluntary apnea and to compare reliability measurements between different calculation methods and between different splenic volume formulas. The main findings of this report are as follows: (a) baseline splenic volume and apnea-induced volume indicated excellent test–retest reliability between repeated tests, (b) assessment of different baseline volume calculation methods showed that ‘two consecutive maximal measures’ and ‘mean of all measures’ displayed the highest reliability between repeated tests and (c) Pilström formula (1) displayed slightly higher reliability between repeated tests compared to the Koga formula (3), but similar reliability compared to the prolate ellipsoide formula (2) and high reliability and agreement between the two formulas.

Our data indicate high accuracy and consistency in splenic measures during an apnea test of maximal voluntary effort, assessed by ultrasonography between two identical test situations. Specifically, ICCs of resting splenic volume displayed excellent intra-rater reliability, showing that ultrasonography-derived splenic volume at rest is consistent on repeated measurements, highlighted by strong agreement and correlation of measurements between tests, as calculated by ‘two consecutive maximal measurements’ and ‘mean of all measures’. This is in agreement with previous reports by Hosey et al.^10^, who also found high intra-rater reliability. However, their assessments are only based on the length and width of the spleen, as opposed to a calculated splenic volume. Further, the CV% of the baseline volume was also notably low, indicating low within-subject variability between test occasions. Expressed in practical terms, the splenic volume will vary with 4.5 to 6.4 mL on repeated measurements, using ‘two consecutive maximal measures’ and ‘mean of all measures’ using formula (1), which is notably low concidering normative splenic volumes^11,13^. On the other hand, ‘one single measure’ displayed lower consistency between test occasions, as shown by moderate to good intra-rater reliability and higher CV%, with TE of ~ 15 mL, for all formulas. This increased variability of the ‘one single measure’ method, that only takes one value from the five minutes before the apnea as a representative baseline splenic volume, seems logical, as the spleen is highly vascularized with a continuous pulsating blood flow^1^ resulting in high-frequency changes in volume throughout the rest period. Consequently, our data indicate that ‘two consecutive maximal measures’ and ‘mean of all measures’ are more reliable and consistent compared to ‘one single measure’ and therefore represent a more appropriate method to establish a resting baseline splenic volume value. Which of the two methods to employ, should be based upon individual volume variations during the baseline splenic measurement period. We found a CV of between 1.1 to 2.2% between the two-volume measures used as the ‘two consecutive maximal measures’ method, therefore we suggest that a CV of approximately 3% or less, should be sought after if this particular method should be implemented, as a means to increase reliability. The rationale for the ‘two consecutive maximal measures’ method is that it may limit variation brought about by either the pulsatile changes in volume due to continuous blood flow (biological variation) and/or minor differences in probe placements (measurement error) between collections. Consequently, two similar volume measurements that come in succession with a one-minute interval would likely reflect a more valid baseline volume, effectively excluding values due to pulsatile blood flow or varying probe placements. However, if the volume variation is too great and the two-volume measurements no longer are similar to one another, i.e. with increased variation, this assumption is violated and the ‘mean of all measures’ method should preferably be employed.

In addition, the apnea-induced splenic volume exhibited similar, excellent intra-rater reliability and low within-subject variability, indicating that this consistency of splenic volumes assessed by ultrasound, is similar also in response to a specific stressor, suggesting that the magnitude of splenic contraction induced by hypoxia is consistent on repeated measurements. Therefore, splenic volumes obtained by ultrasonic imaging have practical applications during apnea, as a method detecting splenic volume change consistently between repeated measurements. In contrast to clinical research, which is more inclined towards the determination of normative splenic volumes to accurately diagnose various symptoms and disorders. Physiological research is predominantly interested in the assessment of magnitudes of responses induced by a stressor or by measurements between groups. Consequently, it is vital to, with high accuracy, replicate measurements consistently between repeated measurements and between groups. Our data indicate that splenic volumes recorded by ultrasonography are highly reliable with low variation, which has practical applications during physiological laboratory and field-based investigations involving various stressors, including hypoxia.

The splenic length measured by ultrasound is generally strongly correlated with the actual splenic volume of cadavers^20^ and with measurements derived by CT^14,21^, indicating that splenic volumes recorded by ultrasonography are valid representations of the actual splenic size. Further, splenic volume change in response to apnea has been assessed by MR imaging, which, in line with splenic volume change assessed by ultrasound, also displays a significant apnea-induced splenic contraction^15^. The conventional method to calculate splenic volume from the three diameters is typically formula (2), which applies a function of 0.524^11,13^ and have also, indirectly, been validated^14^. Koga^23^ was the first study to calculate splenic volume using ultrasonography, however, this formula is limited to only include two splenic dimensions and estimating splenic volume based on the sectional area, without any information regarding splenic width, typically measured in the transverse plane. Therefore, it is highly plausible that formula (3) over or underestimates splenic volumes of individuals with particularly atypical splenic shapes. Our data indicate that formula (3) has reduced consistency between repeated tests, pertaining to both CV% and ICC, for each baseline calculation method compared to both formula (1) and formula (2). One likely reason for this slightly lower reliability of formula (3) could likely be ascribed to the fact that it does not include triaxial measurements and thereby are less representative of individual variations in splenic shapes.

Formula (1)^8,17,30^, was designed in response to individual variation in splenic diameters^10,11^, which potentially could result in inconsistent splenic volumes. Our data highlights minor differences in splenic volume between formulas. By viewing Fig. 4C,D it is quite clear that the differences between formula (1) and formula (2) are small within each participant for both ‘two consecutive maximal measures’ and ‘mean of all measures’, denoted by a small systematic measurement error (bias), wherein none of the participants in the sample falls outside the limits of agreements, displaying high agreement. Second, it is also clear, that the calculated splenic volumes for both formulas are not consistently higher or lower than the other. Thus, one of the formulas does not overestimate or underestimate volumes derived by the other formula. Instead, different splenic volumes likely depend on specific measures of individual splenic diameters. This may be further substantiated by Fig. 5 that shows variations in splenic volumes between formulas as a function of varying diameters, wherein splenic width likely leads to a greater degree of volume variations. This may suggest that formula (1) may account for individual differences in splenic dimensions in a dynamic way and therefore reflect varying splenic shapes. Although the agreement between formula (3) and formula (1) was good at best, with a systematic measurement error within 15 mL, it was lower compared to the agreement between the formulas including triaxial measurements.

Nevertheless, even though the differences in splenic volumes between formulas are relatively small, displaying good to excellent ICCs and minor CV%, which indicates high reliability, the accuracy of the measurement error, as assessed by 95% limits of agreements, was relatively low, particularly between formula (1) and formula (3). This low accuracy of the measurement error can potentially result in substantial differences in splenic volumes between formulas and should be taken into account when comparing splenic volumes across studies using different formulas. However, as all formulas display high ICCs and low within-subject variation in baseline splenic volume across repeated measurements, comparisons between splenic response magnitudes are highly relevant between formulas, but comparisons of absolute splenic volume values should preferably be avoided.

## Conclusion

We conclude that both baseline splenic volume and the apnea-induced volume assessed by ultrasonography, has excellent reliability between repeated tests for both the Pilström formula and the prolate ellipsoid formula, suggesting that ultrasonographic measurements of the spleen have practical application during laboratory and field-based research involving apnea and hypoxia, as a reliable method detecting splenic volume change, consistently between repeated measurements. Additionally, to determine an accurate baseline splenic volume, our data highlights two methods: (1) averaging the two consecutive maximal volume measurements during a five-minute resting period and (2) obtaining an average of all volume measurements during a five-minute resting period. Determination of which of the two methods to implement should preferably be assessed based on individual volume variation between the selected two maximal values. The Pilström formula exhibited slightly higher reliability compared to the Koga formula, but similar reliability and variability compared to the prolate ellipsoid formula, with a small systematic measurement error showing high agreement, suggesting that the Pilström formula may be particularly useful to account for individual differences in splenic dimensions. However, the accuracy of the measurement error was low, consequently, comparisons of absolute splenic volumes between studies using different formulas should be avoided.

## Supplementary Information

Supplementary Information.

## Data Availability

All data generated or analysed during this study are included in this published article.

## Abbreviations

MRI: Magnetic resonance imaging
CT: Computed tomography
ICC: Intraclass correlation coefficient
CV: Coefficient of variation
LoA: Limits of agreement
RBC: Red blood cells
yrs: Years
cm: Centimeters
kg: Kilograms
m: Meter
SpO_2_: Peripheral oxygen saturation
L: Splenic length
T: Splenic thickness
W: Splenic width
$$V_{spleen}$$: Spleen volume
SD: Standard deviation
CI: Confidence interval
TE: Typical error
mL: Milliliter
